# Passive Acoustic Monitoring the Diel, Lunar, Seasonal and Tidal Patterns in the Biosonar Activity of the Indo-Pacific Humpback Dolphins (*Sousa chinensis*) in the Pearl River Estuary, China

**DOI:** 10.1371/journal.pone.0141807

**Published:** 2015-11-18

**Authors:** Zhi-Tao Wang, Paul E. Nachtigall, Tomonari Akamatsu, Ke-Xiong Wang, Yu-Ping Wu, Jian-Chang Liu, Guo-Qin Duan, Han-Jiang Cao, Ding Wang

**Affiliations:** 1 The Key Laboratory of Aquatic Biodiversity and Conservation of the Chinese Academy of Sciences, Institute of Hydrobiology, Chinese Academy of Sciences, Wuhan, P. R. China; 2 University of Chinese Academy of Sciences, Beijing, P. R. China; 3 Marine Mammal Research Program, Hawaii Institute of Marine Biology, University of Hawaii, Hawaii, United States of America; 4 National Research Institute of Fisheries Engineering, Fisheries Research Agency, Ibaraki, Japan; 5 School of Marine Sciences, Sun Yat-Sen University, Guangzhou, P. R. China; 6 Transport Planning and Research Institute, Ministry of Transport, Beijing, P. R. China; 7 Hongkong-Zhuhai-Macao Bridge Authority, Guangzhou, P. R. China; Virginia Commonwealth University, UNITED STATES

## Abstract

A growing demand for sustainable energy has led to an increase in construction of offshore windfarms. Guishan windmill farm will be constructed in the Pearl River Estuary, China, which sustains the world’s largest known population of Indo-Pacific humpback dolphins (*Sousa chinensis*). Dolphin conservation is an urgent issue in this region. By using passive acoustic monitoring, a baseline distribution of data on this species in the Pearl River Estuary during pre-construction period had been collected. Dolphin biosonar detection and its diel, lunar, seasonal and tidal patterns were examined using a Generalized Linear Model. Significant higher echolocation detections at night than during the day, in winter-spring than in summer-autumn, at high tide than at flood tide were recognized. Significant higher echolocation detections during the new moon were recognized at night time. The diel, lunar and seasonal patterns for the echolocation encounter duration also significantly varied. These patterns could be due to the spatial-temporal variability of dolphin prey and illumination conditions. The baseline information will be useful for driving further effective action on the conservation of this species and in facilitating later assessments of the effects of the offshore windfarm on the dolphins by comparing the baseline to post construction and post mitigation efforts.

## Introduction

Marine mammal species occurring in coastal areas are the most likely ones to be at-risk from anthropogenic effects [1]. The Indo-Pacific humpback dolphins (*Sousa chinensis*, locally called the Chinese white dolphin) was categorized as Near Threatened by the International Union for the Conservation of Nature Red List of Threatened Species [2]. It’s widely distributed throughout the shallow, coastal waters from the Southern China Sea in the east to the eastern India in the west and throughout Southeast Asia [2, 3]. This species has a general preference for estuarine habitats, recognized as transitional zone linking fresh- and marine-water [4], however, the coastal distribution of this species make them susceptible to the impact of human activity [1].

**Fig 1:**
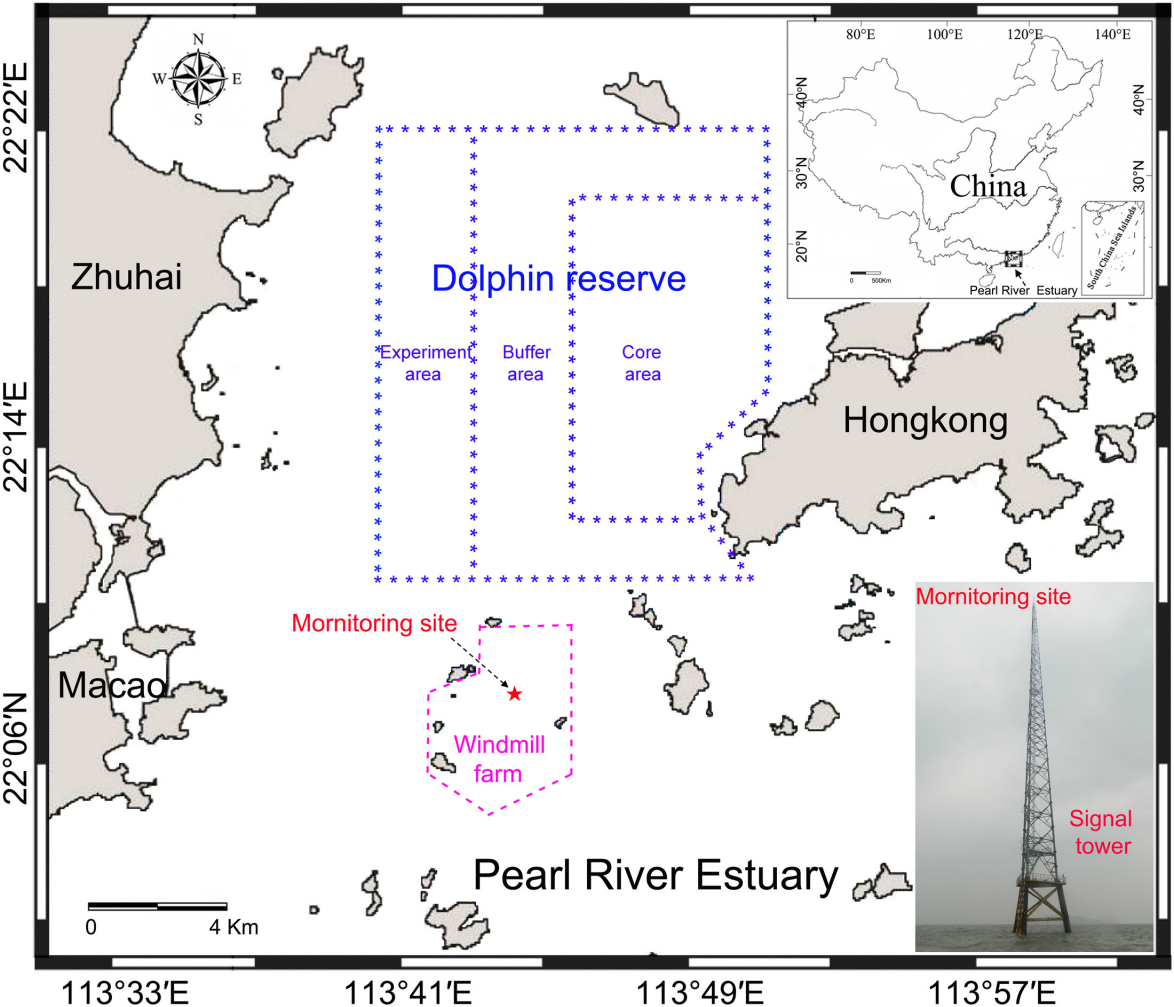
Map of the static acoustic monitoring area. Passive acoustic monitoring device was deployed below a signal tower. The purple dash line shows the area of the planned windmill farm, the blue line area indicates the national Chinese white dolphin nature reserve. The inset map in the lower right corner shows the signal tower.

The Pearl River Estuary (Fig 1) is located in subtropical areas of the northern part of the South China Sea, which sustains the world’s largest known population of humpback dolphins [5, 6], with the population size estimated to be over 2500 (CVs: 19–89%) [5]. The area is among the most economically developed regions in China [7] and economic growth has been accelerating human impact on coastal ecosystems [8]. A growing demand for environmentally friendly and sustainable energy has led to an increase in construction of offshore windfarms and the Guishan windmill farm was recently licensed within the Linding waters of the Pearl River Estuary. The location is about 2 km south of the national Chinese white dolphin nature reserve, 15 km southeast of Zhuhai and 10 km east of Macao. This windfarm project consists of a transformer platform and 66 wind turbines with a rated power of 3 MW each arranged in 8 rows and covers an area of about 32.6 km^2^ with a water depth between 6 and 12 m. Construction and operation of the wind farm potentially affects aquaticlives and might cause marine mammals avoidance of the developing area [9]. The considerable noise emissions associated with pile driving during windfarm construction can cause acoustic disturbance [10] and/or behavioral disruption at ranges of many kilometers [11] and may potentially harm marine mammals in the vicinity by causing temporal or permanent hearing threshold shifts [12] or physical injury [13]. Prolonged disturbance may induce animals to move away, temporarily or permanently, and this may expose the population to unknown, potentially unfavorable, new environmental conditions (e.g. lower food resources, unknown dangers, etc.) to which they are not adapted [13]. In order to protect the humpback dolphins, baseline data during the pre-construction period on the dolphin distribution and time-specific habitat preferences in the construction area is urgently required. With modelization of the propagation range of generated noise and of levels to be expected in the protected area, these baseline data can help in assessing the effects of offshore wind farms on the animals as well as to designing and enforcing effective mitigation programs.

Marine mammals, especially cetaceans, have evolved sophisticated sound production mechanisms and rely primarily on their acoustic sensing biosonar for communication, navigation and foraging [14, 15]. Beside emitting whistles with a mean fundamental frequencies of 6.4 kHz, and minimum and maximum fundamental frequency averaged at 5.1 kHz and 7.7 kHz, respectively [16], humpback dolphins also produce pulsed sound with a mean peak-peak source level of 199 ± 3 dB re 1μPa at 1m, and mean centroid and peak frequency of 106 ± 11 kHz and 114 ± 12 kHz, respectively [17]. Toothed whales (Odontoceti) generally use biosonar in an intense manner, such as the harbor porpoises (*Phocoena phocoena*) and Yangtze finless porpoise (*Neophocaena asiaeorientalis asiaeorientalis*) that produce sonar click trains every 12.3 and 6.4 s on average) [18], which facilitate the acoustic monitoring of these animals. Passive acoustic monitoring is a well-established and rapidly evolving method to obtain information on the occurrence, distribution, relative abundance and acoustic behavior of many aquatic mammals over a range of spatial and temporal scales [19, 20]. This method is able to be applied during rough weather conditions and during poor visibility, such as at nighttime periods [21], which is logistically impossible for visual observations. Additionally the observed data can be independent of human observer limitations and biases.

A previous line transect survey based on visual means, combined with photo-identification studies suggested seasonal variations in distribution of some individual humpback dolphins in the Pearl River Estuary [22, 23], however, detailed information on the temporal trends in the occurrence of dolphin in this region has not been determined. This shortcoming constrains our understanding of whether the expansion of marine renewables poses a significant threat to the local dolphins and should be addressed as a priority.

By using static acoustic monitoring, the principal objective of this study was to describe the presence of humpback dolphin in the windmill site of the Pear River Estuary during the pre-construction period to fill the knowledge gap. The potential influence of time of the day, lunar, seasonal and tidal phases on their biosonar behavior was further examined.

## Methods

Static acoustic monitoring was carried out from a mobile phone signal tower (22°07′54″ N, 113°43′54″ E) located within the planed area of the Guishan windmill farm (Fig 1) during December, 2013 to August, 2014 (Table 1). The acoustic recording system was attached on a steel wire rope and suspended below the signal tower in the middle of water column (4.0 m above the ocean floor and approximately 3.0 to 5.8 m below the water surface depending on the tide). A 30 kg anchor block was attached on the bottom side of the steel wire rope and laid down on the seabed to reduce the movement of the recording system due to water currents. Geophysical studies indicated that the bottom topography is smooth. The substrate consists of mainly silt-clays, sand, and gravel (TQ Zeng, personal communication).

**Table 1:** Deployment schedule and detections of humpback dolphin biosonar sound. Number of click trains and buzzes and minutes with click trains and buzzes, and echolocation encounters (including encounter numbers, encounter group size, and duration) were summarized for each trial. The duration of echolocation encounters is given as the mean ± standard error of the mean (SEM) and range. Numbers in parentheses indicate the number of echolocation encounters with group number over than two.

Date		Duration(days)	Number of click trains	Number of minutes with click trains	Number of buzzes	Number of minutes with buzzes	Echolocation encounter duration(min)				
Deployment	Retrieval						N	Mean	SEM	Min	Max
12/25/2013	1/03/2014						62(7)	12.61	2.30	0.001	86.10
1/06/2014	1/17/2014	12	677	314	79	54	61(5)	9.68	1.68	0.007	70.50
1/26/2014	1/31/2014	6	906	431	117	100	45(5)	16.04	2.86	0.002	65.40
2/26/2014	3/12/2014	15	435	190	23	19	38(2)	8.11	2.24	0.007	63.60
3/16/2014	3/26/2014	11	616	255	63	41	45	8.40	1.62	0.002	53.60
4/13/2014	4/26/2014	14	151	82	23	14	28	4.40	1.27	0.006	31.92
5/26/2014	6/03/2014	9	96	40	3	3	13	4.02	1.02	0.004	9.80
6/04/2014	6/13/2014	10	216	106	12	9	21	7.34	1.41	0.003	21.69
6/17/2014	6/25/2014	9	47	24	6	5	4	8.34	4.46	0.002	17.07
7/02/2014	7/10/2014	9	58	34	3	3	17	2.04	0.84	0.001	11.03
7/11/2014	7/22/2014	12	96	49	2	2	16	3.30	1.36	0.007	22.12
8/01/2014	8/10/2014	10	172	83	12	6	23	5.00	1.38	0.003	23.09
Total		127	4432	2026	561	384	373(19)	8.79	0.68	0.001	86.10

### Passive acoustic monitoring system

Acoustic data logger, named A-tag (ML200-AS2, Marine Micro Technology, Saitama, Japan) were used in this study to monitor the biosonar sounds of the dolphins. An A-tag is a self-contained and submersible data logger designed to detect and record ultrasonic pulse events. Each A-tag consisted of two ultrasonic hydrophones (primary hydrophone, the one closer to the processing unit of the A-tag and a secondary hydrophone, the one further away from the body of the A-tag), a band-pass filter (-3 dB with a 55kHz -235 kHz range), a high-gain amplifier (+60 dB), a 128 MB flash memory card and two UM-1 batteries (LR20) [24]. The hydrophones (model MHP-140ST; Marine Micro Technology, Saitama, Japan) formed a stereo hydrophone array by separating at a fixed range of either 195 mm (short baseline) or 590 mm (long baseline). The primary and secondary hydrophones had identical sensitivities of -201 dB re 1 V/μPa at 1 m distance and a frequency response range from 100 Hz to 160 kHz plus and minus 5 dB. The sampling interval of the A-tag was either 0.5 ms (short baseline: corresponding to sample rate of 2000 Hz) or 2 ms (long baseline: corresponding to sample rate of 500 Hz). Within each sampling interval, once a hydrophone was triggered by a pulse sound that surpassed a preset hardware detection threshold level, a high-speed counter would wait for the trigger arriving at the other hydrophone and measure the time of arrival difference between the stereo hydrophones with a resolution of 271 ns (short baseline) or 1084 ns (long baseline), in detail, if the primary hydrophone was first triggered, a positive time difference was logged, if the secondary hydrophone was first triggered, a negative time difference was logged. In order to prevent the A-tag’s memory from being filled up by background noise and to save battery, only the information about the ultrasonic pulse events including the absolute detection time of a sound pulse, peak sound pressure levels detected by both hydrophones, and the difference in arrival time were recorded instead of recording the signal raw waveforms. The dynamic range of the A-tag was 129 dB to 160 dB peak-peak re 1 μPa with an internal thermal noise of approximately 134 dB. The high thermal noise level wasdue to the contamination of thermal and CPU clock noise into the hydrophone code. Initial +60dB amplification of these noises continuously trigger A-tag when the detection threshold level was set too low. Therefore we employed high detection threshold level comparing with the natural background noise. In this study, a hardware detection threshold of 135.7 dB was adopted by the A-tag to standardize the dataset.

### Diel phase classification

**Fig 2:**
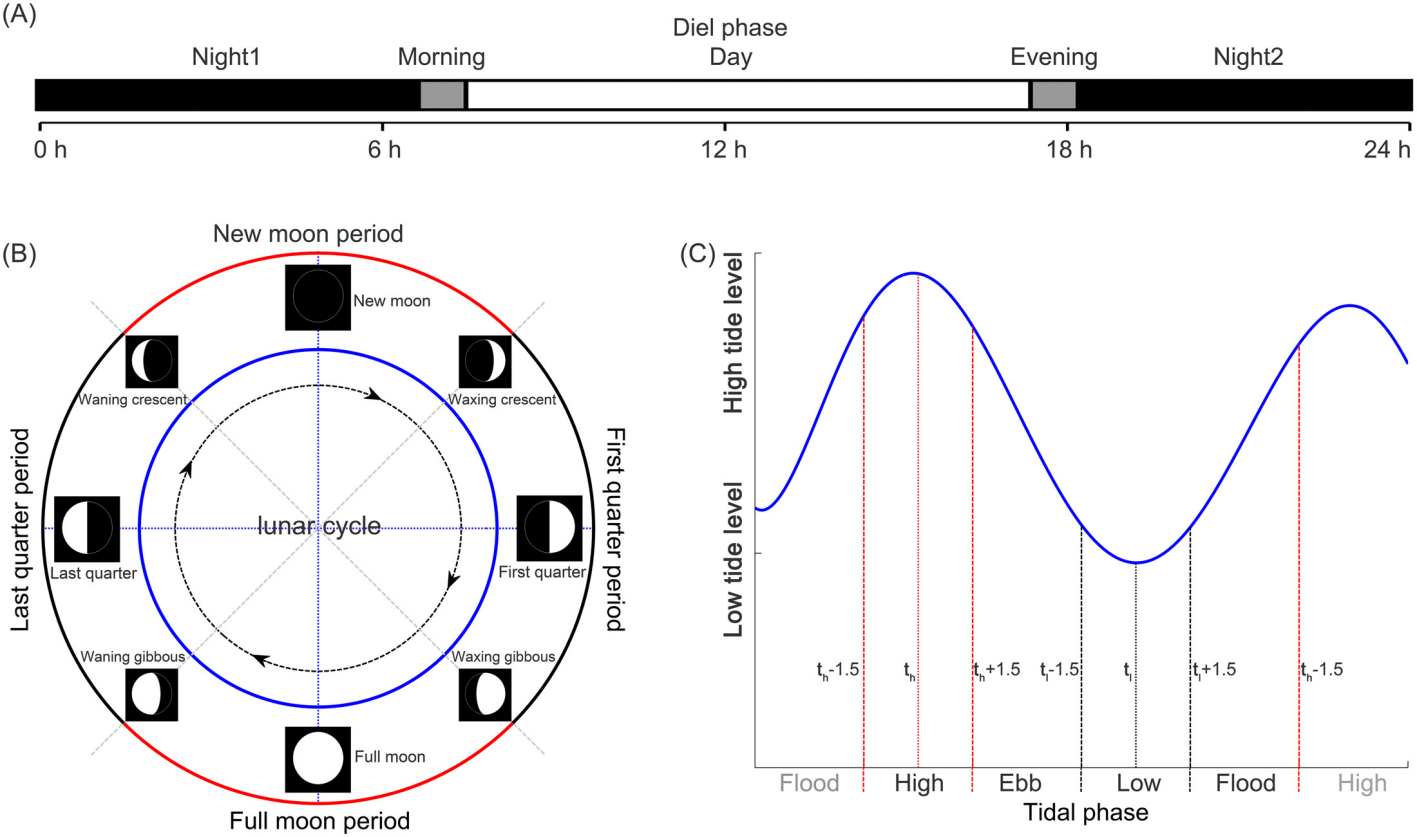
Schematic of the assignment of (A) diel, (B) lunar, and (C) tidal phases. Diel phase was divided into night1, morning, day, evening, and night2. Lunar period was composed of new moon, first quarter, full moon, and last quarter periods. Tidal phase was composed of high, ebb, low, and flood phases. t_h_ and t_l_ represent the time point when the highest and lowest water level, respectively.

The 24 h of the day were divided into five consecutivediel phases in sequence of night1, morning, day, evening and night2 [25] (Fig 2A) according to the equations [26]:

civil twilight start ≤ morning ≤(2sunrise−civil twilight start) (1)

(2sunset − civil twilight end) ≤ evening ≤ civil twilight end (2)

where civil twilight start and civil twilight end referred to the time point when the center of the sun was geometrically 6° below the horizon with a trend of decrease and increase in the degree between the center of the sun and the horizon, respectively. Sunrise and sunset referred to the times when the upper edge of the sun was on the horizon. Night1 and night2 were separated at 12:00 pm (Fig 2A). Eachtime point at the location of the acoustic monitoring siteduring the study periods were obtained from the web site of the US Naval Observatory (http://aa.usno.navy.mil).

### Lunar period classification

The period of the lunar cycle was fixed a-priori at 29.53 days [27, 28]. Based on the four primary phases of the moon (in the sequence of their occurrence): new moon (the moon is not visible with its unilluminated side facing the earth), first quarter (one-half of the moon appears to be illuminated with the illuminated fraction of the moon's disk showing a trend of increasing), full moon (the moon appears to be completely illuminated with its illuminated side facing the earth) and last quarter (one-half of the moon appears to be illuminated with the illuminated fraction of the moon's disk showing a trend of decreasing) (Fig 2B), lunar cycle was divided into the following four consecutive periods: new moon period (foothold at the phase of new moon and backward to halfway to the former last quarter phase and forward to halfway to the later first quarter), first quarter period (foothold at the phase of first quarter and backward to halfway to the former phase of new moon and forward to halfway to the later phase of full moon), full moon period (foothold at the phase of full moon and backward to halfway to the former phase of first quarter and forward to halfway to the later phase of last quarter) and last quarter period (foothold at the phase of last quarter and backward to halfway to former phase of full moon and forward to halfway to the later phase of new moon) (Fig 2B). The time point for four primary phases of the moon, including new moon, first quarter, full moon and last quarter were obtained from the web site of the US Naval Observatory (http://aa.usno.navy.mil).

### Seasonal classification

Seasons were defined by the four quarters of the year (January-March: Spring; April-June: Summer; July-September: Autumn; October- December: Winter). According to the seasonal precipitation intensity variations, summer and autumnwere also called wet seasons, whereas winter and spring were also called dry seasons.

### Tidal phase classification

The tidal period in the Pearl-River Estuary are semi-diurnal (12^+^hour) and the typical tidal ranges (defined as the difference in water level between high water and low water) are 0.8 m at neap tides and 2.8 m at spring tides. The tidal condition was divided into four consecutive phases: low, flood, high, and ebb. Low and high phases were from the one and a half hours before to the one and a half hours after the high tide and low tide, respectively [29, 30]. Flood and ebb phase were the period between low phase to high phase and high phase to low phase, respectively. The time point for the high tide and low tide were obtained from the web site of the China Shipping Service (CNSS) (http://ocean.cnss.com.cn/).

### Acoustic data analysis

**Fig 3:**
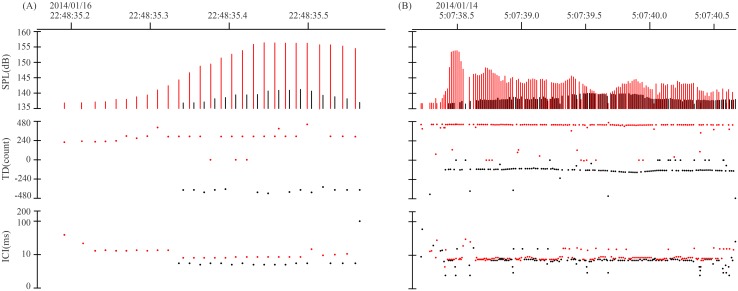
Ultrasonic pulse events with two click trains overlapped. The two click trains were from two different direction (red and black trace corresponds to the direction to the primary hydrophone and the secondary hydrophone sides of the A-tag, respectively). The top panel shows the received peak sound pressure level (SPL, dBre: 1μPa peak-peak) of each sound pulse, middle panel shows the time difference (TD, resolution of 271 ns or 1084 ns for short or long baseline A-tag, respectively) of the same sound pulse arrived at the two hydrophones of the A-tag, and the bottom panel shows the inter click intervals (ICI) within the click trains. Note that the temporal scale in (A) and (B) were different.

Upon retrieval of the recorder, acoustic data were downloaded and processed during the off-line signal processing by using two step procedures to filter out non-dolphin clicks and extract the dolphin biosonar signals. A custom-made multi-parameter filter program based on Igor Pro 5.01 (Wave Metrics, Lake Oswego, OR, USA) was adopted during the pilot trial to classify the recorded pulses as originating from dolphins, boats or other sound sources [31]. The discriminating parameters included the minimum number of pulses in a click train, the maximum duration and the differences in inter click interval between 2 adjacent pulses. Here, the minimum number of pulses in a click train was conservatively set at 5 [18, 25, 31] since dolphin sonar signals nearly always exhibits as train of pulses [32]. Successive clicks were considered as one click train if the inter-click interval was shorter than 200 ms [33–35]. In addition, dolphin click trains were identified as having smoothly changing patterns of inter-click intervals, with successive inter-click intervals greater than half and less than twice the previous ones [36]. Pulses within 2 ms after the detection of the direct path pulse, corresponding to the sampling interval of the long baseline A-tag, were eliminated as possible surface or bottom reflections (for reasons see discussion section). During the second step, possible click trains of dolphins detected by the offline filter were visually confirmed twice to eliminate spurious detections such as ship noise or snapping shrimp sounds. Dolphin click trains were identified as having smoothly changing patterns of sound pressure levels and inter-click intervals [36], this characteristic can aid in distinguishing false detections originating from noise, which characterized randomly changing sound pressure levels and inter click intervals [31]. Short-range sonar sounds with minimum inter-click intervals shorter than 10 ms, often called buzzes have been widely used as indicators of attempts at prey capture [37], feeding activity [26, 38] or foraging success [39, 40] were also noted and treated as a proxy indication of feeding activity [25, 31]. An echolocation encounter was defined as a group of click trains separated by 10 minutes or less [26, 38]. Thus, if click trains were separated by more than 10 minutes, they were considered to be different echolocation encounters. Echolocation encounter duration was calculated by subtracting the start day and time of a single echolocation encounter from the end day and time of that same echolocation encounter. In some cases, click trains from two dolphins from different direction was observed (Fig 3). This pattern could be used for counting the group size of each echolocation encountersin addition to the numbers of independent sound source directions. Here the group size was estimated as either single or ≥ two.

All the acoustic data was partitioned into non-overlapping 10 minute bins, which always began on the hour to account for the difference in sampling effort across diel, lunar, seasonal and tidal phases. The time boundaries for different diel, lunar and tidal phases were also rounded to the nearest 10 minute bin. Each echolocation encounter was also assigned to appropriate phases. For the analysis of diel, lunar, seasonal and tidal pattern, echolocation encounters that spanned multiple periods were segmented, and each segment was assigned an appropriate diel, lunar, seasonal or tidal period. During number counting, echolocation encounters that crossed boundaries between periods were assigned to the dominant period that contained the greater portion. Four parameters of dolphin biosonar behavior per 10 minutes were employed, such as the number of click trains, number of minutes with click trains, the number of buzzes and number of minutes with buzzes. The first two parameters indicate the sensing effort of dolphins and the latter half of the buzz related parameters indicate the feeding activity of dolphins within the detection range of the acoustic recorder.

### Statistical analysis

Descriptive statistics (mean, standard error of the mean (SEM), and range) were used to summarize biographical information. All the parameters were tested for normality and homoscedasticity. Data sets smaller than 50 samples were tested for normality using the Shapiro–Wilk test; larger datasets with over 50 samples were analyzed with the Kolmogorov–Smirnov test. Levene's test for equality of variance was used to analyze the homogeneity of the variance. For the analysis of the difference of the biosonar parameters as a function of diel, lunar, seasonal and tidal conditions, the generalized linear model (GLM) analysis of variance (ANOVA) procedure was applied with four-way ANOVA (diel * lunar * season * tidal) full factorial design by including diel, lunar, season and tidal into the model as main factors and building interaction terms into the model. When significant differences were found for either main factor, more focused analysis of the post-hoc pair-wise multiple comparison tests were performed using the Tukey’s HSD method when the Levene's test indicated homogeneous variances (*P* > 0.05); otherwise, Tamhane's T2 method was applied if equal variances could not be assumed (*P*< 0.05) to probe which levels of each factor significantly differed. For the analysis of whether or not significant differences existed for the echolocation encounter duration as a function of diel, lunar, seasonal or tidal conditions, if data sets were normally distributed (*P*> 0.05), one-way ANOVA was applied to test for the overall difference and further analyzed with either Tukey’s HSD post hoc test (equal variances; *P*> 0.05) or Tamhane’s T2 post hoc test (variances not equal; *P*< 0.05) when *P*< 0.05 by one-way ANOVA to determine how the parameter varied among different diel, lunar, seasonal or tidal conditions. When data sets were not normally distributed (*P*< 0.05), the Kruskal–Wallis nonparametric tests was used and further analyzed with Duncan's multiple comparison test [41] where applicable (Kruskal–Wallis test; *P*<0.05). Statistical analyses were performed using SPSS 16.0 for Windows (SPSS Inc., Chicago, IL, USA). Probability values exceeding 0.05 was considered to be the critical statistical level of significance.

### Ethical statement

Permission to conduct the study was granted by the Ministry of Science and Technology of the People’s Republic of China. The research permit was issued to the Institute of Hydrobiology of the Chinese Academy of Sciences (Permit number: 2011BAG07B05). No disturbance to dolphins was produced during the experiments.

## Results

**Fig 4:**
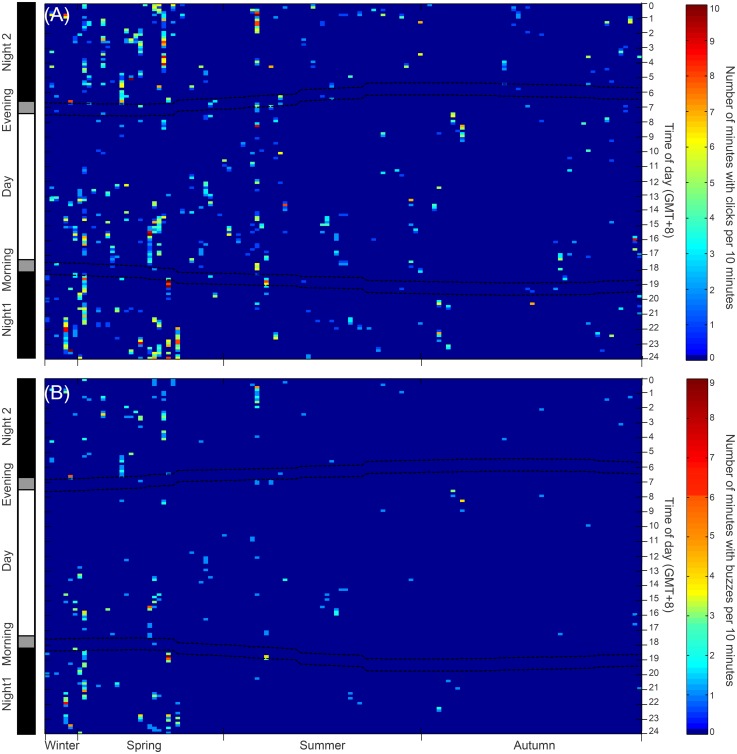
The daily occurrence of (A) dolphin click trains and (B) buzzesas a function of time of day and seasonal conditions. The dashed broken lines indicate the boundary of different dial phases. Times are given as local standard time (UTC + 8 hr).

Over the 127 recording days, which included over 16759 ten minutes bins of acoustic data, 4432 click trains of humpback dolphin were identified. In total, 684 ten minutes recording bins (4.1% of all ten minutes recording bins) contained echolocation encounters. A total of 99 recording days (77.9% of all recording days) included at least one positive time bin with dolphin sonar in a day (Fig 4, Table 1).

Five hundred and sixty one buzzes of humpback dolphin were identified. In total, 213 ten minutes recording bins (1.4% of all ten minutes recording bins) contained echolocation buzzes. A total of 62 recording days (48.8% of all recording days) included at least one positive time bin with dolphin buzzes in a day (Fig 4, Table 1). 373 echolocation encounters were identified and 5.1% of them classified as groups with two or more individuals (19 out of all echolocation encounters). The average duration of echolocation encounter was 8.79 ± 0.68 minutes, with the minimum and maximum duration of 0.001 and 86.10 minutes, respectively (Fig 4, Table 1).

### Diel patterns

**Fig 5:**
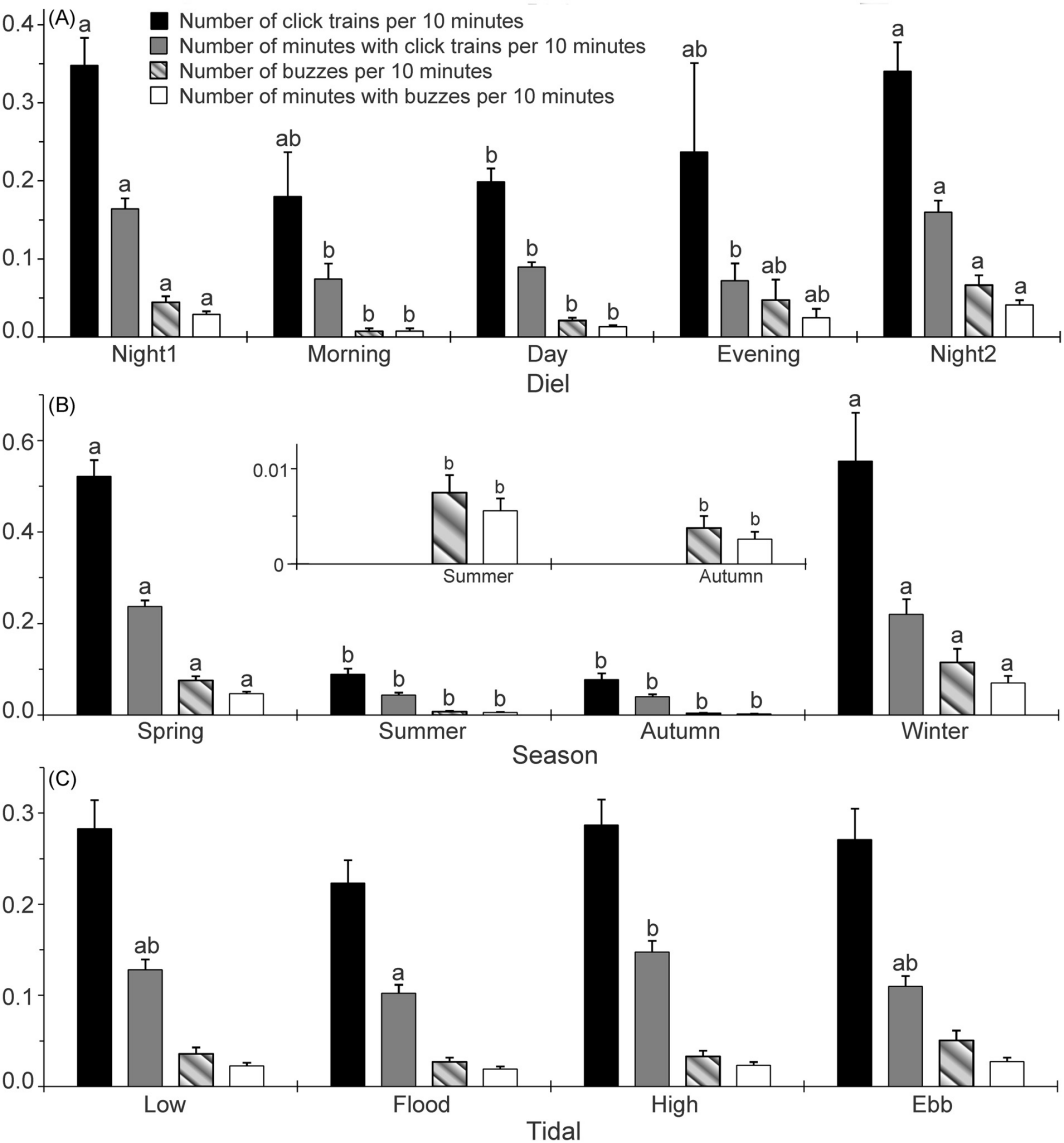
Bar graphs of the number of click trains, number of minutes with click trains, number of buzzes and number of minutes with buzzes per 10 minutes as a function of (A) Diel, (B) seasonal and (C) tidal phases. Results are expressed as mean ± standard error of the mean (SEM), Error bars with different lowercase letters refer to Tamhane’s T2 post hoc multiple-comparison test that yielded significant results *P*< 0.05.

**Fig 6:**
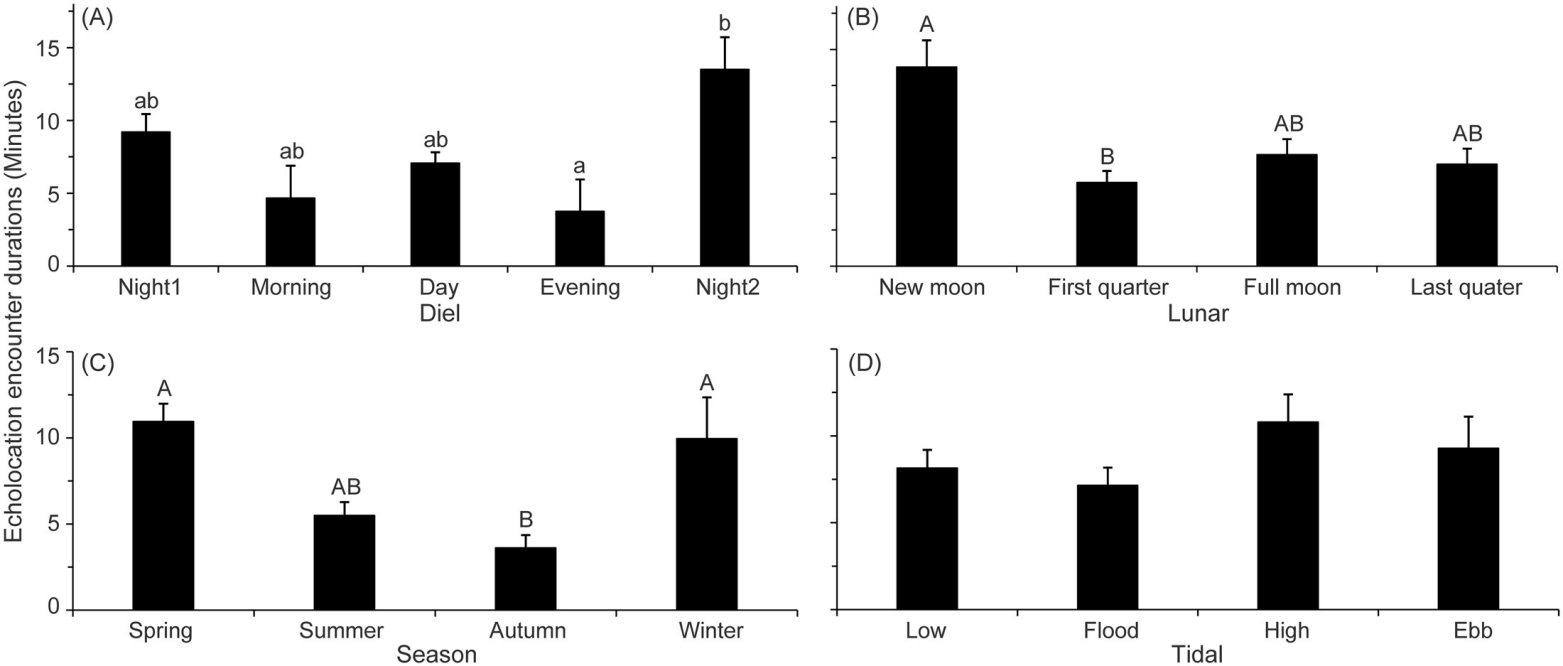
Echolocation encounter duration as a function of (A)Diel, (B)lunar, (C)season, and (D)tidal phase. Error bars (mean ± SEM) with different uppercase and lowercase letters refer to post hoc Duncan’s multiple-comparison tests that yielded significant results at *P*< 0.01 and *P*< 0.05, respectively.

The results of GLM ANOVAindicate that significant differences in diel patterns exist in all the parameters, the number of click trains per 10 minutes (Table 2), the number of minutes with click trains per 10 minutes (Table 3), the number of buzzes per 10 minutes (Table 4), and the number of minutes with buzzes per 10 minutes (Table 5). In particular, the number of click trains per 10 minutes was significantly higher during night1and at night2 than that during the day (Tamhane’s T2 post hoc pairwise multiple comparison tests; *P*< 0.05) (Fig 5A). The number of minutes with click trains per 10 minutes at night1 and at night2 were significantly higher than that in the morning, during the day, and in the evening (Tamhane’s T2 post hoc multiple-comparison test; *P*< 0.05) (Fig 5A). Both of the number of buzzes per 10 minutes and number of minutes with buzzes per 10 minutes at night1 and at night2 were significantly higher than that in the morningand during the day (Tamhane’s T2 post hoc multiple-comparison test; *P*< 0.05) (Fig 5A). Whereas no significant differences were observed among at night1, at night2, and in the evening (Tamhane’s T2 post hoc multiple-comparison test; *P*> 0.05) (Fig 5A). Significant diel patterns were also observed in the echolocation encounter duration (Kruskal-Wallis χ^2^ = 11.11, df = 4, *P* = 0.03). In particular, the echolocation encounter duration was significantly shorter in the evening than that at night2 (Duncan's multiple-comparison test; *P*< 0.05) (Fig 6A).

**Table 2:** Results of four-way ANOVA (diel * lunar * season * tidal) on the number of click trains per 10 minutes. The main effects of diel and season, and the interaction effects of diel * lunar, diel * season, diel * tidal, lunar * season, season * tidal, diel * lunar * season, diel * lunar * tidal, diel * season * tidal, lunar * season * tidal and diel * lunar * season * tidal were all significant sources of variability in the occurrence of click trains per 10 minutes. Bold numbers indicate significant effects (*P*< 0.05).

Source	Type III Sum of Squares	df	Mean Square	F	*P*
Corrected Model	3806.68	218	17.46	4.90	0.000
Intercept	169.23	1	169.23	47.48	0.000
Diel	45.25	4	11.31	3.17	**0.013**
Lunar	8.14	3	2.71	0.76	0.516
Season	257.16	3	85.72	24.05	**0.000**
Tidal	11.21	3	3.74	1.05	0.370
Diel * lunar	178.12	12	14.84	4.17	**0.000**
Diel * season	83.52	12	6.96	1.95	**0.024**
Diel * tidal	103.84	12	8.65	2.43	**0.004**
Lunar * season	66.51	6	11.09	3.11	**0.005**
Lunar * tidal	35.40	9	3.93	1.10	0.356
Season * tidal	63.57	9	7.06	1.98	**0.037**
Diel * lunar * season	369.22	23	16.05	4.50	**0.000**
Diel * lunar * tidal	297.19	32	9.29	2.61	**0.000**
Diel * season * tidal	193.01	29	6.66	1.87	**0.003**
Lunar * season * tidal	224.37	18	12.47	3.50	**0.000**
Diel * lunar * season * tidal	324.24	41	7.91	2.22	**0.000**
Error	58951.26	16540	3.56		
Total	63930.00	16759			
Corrected Total	62757.94	16758			

**Table 3:** Results of four-way ANOVA (diel * lunar * season * tidal) on the number of minutes with click trains per 10 minutes. The main effects of diel, season and tidal, and the interaction effects of diel * lunar, diel * season, diel * tidal, lunar * season, season * tidal, diel * lunar * season, diel * lunar * tidal, diel * season * tidal, lunar * season * tidal and diel * lunar * season * tidal were all significant sources of variability in the number of minutes with click trains per 10 minutes. Bold numbers indicate significant effects (*P*< 0.05).

Source	Type III Sum of Squares	df	Mean Square	F	*P*
Corrected Model	668.30	218	3.07	6.30	0.000
Intercept	35.70	1	35.70	73.39	0.000
Diel	10.79	4	2.70	5.55	**0.000**
Lunar	1.53	3	0.51	1.05	0.369
Season	37.59	3	12.53	25.76	**0.000**
Tidal	4.46	3	1.49	3.05	**0.027**
Diel * lunar	30.18	12	2.52	5.17	**0.000**
Diel * season	16.44	12	1.37	2.82	**0.001**
Diel * tidal	18.41	12	1.53	3.16	**0.000**
Lunar * season	10.87	6	1.81	3.73	**0.001**
Lunar * tidal	5.28	9	0.59	1.21	0.285
Season * tidal	12.99	9	1.44	2.97	**0.002**
Diel * lunar * season	59.41	23	2.58	5.31	**0.000**
Diel * lunar * tidal	49.57	32	1.55	3.19	**0.000**
Diel * season * tidal	35.41	29	1.22	2.51	**0.000**
Lunar * season * tidal	32.75	18	1.82	3.74	**0.000**
Diel * lunar * season * tidal	41.30	41	1.01	2.07	**0.000**
Error	8044.78	16540	0.49		
Total	8958.00	16759			
Corrected Total	8713.08	16758			

**Table 4:** Results of four-way ANOVA (diel * lunar * season * tidal) on the number of buzzes per 10 minutes. The main effects of diel and season, as well as the interaction effects of diel * lunar, diel * lunar * season, lunar * season * tidal and diel * lunar * season * tidal were all significant sources of variability in the occurrence of buzzes per 10 minutes. Bold numbers indicate significant effects (*P*< 0.05).

Source	Type III Sum of Squares	df	Mean Square	F	*P*
Corrected Model	163.78	218	0.75	3.26	0.000
Intercept	3.90	1	3.90	16.93	0.000
Diel	3.22	4	0.81	3.50	**0.007**
Lunar	1.78	3	0.59	2.57	0.052
Season	5.20	3	1.73	7.52	**0.000**
Tidal	0.65	3	0.22	0.94	0.422
Diel * lunar	5.51	12	0.46	1.99	**0.021**
Diel * season	3.96	12	0.33	1.44	0.142
Diel * tidal	4.02	12	0.34	1.45	0.134
Lunar * season	2.23	6	0.37	1.62	0.138
Lunar * tidal	1.12	9	0.13	0.54	0.845
Season * tidal	3.53	9	0.39	1.71	0.082
Diel * lunar * season	8.35	23	0.36	1.58	**0.039**
Diel * lunar * tidal	6.70	32	0.21	0.91	0.615
Diel * season * tidal	7.14	29	0.25	1.07	0.365
Lunar * season * tidal	12.30	18	0.68	2.97	**0.000**
Diel * lunar * season * tidal	14.10	41	0.34	1.49	**0.022**
Error	3808.79	16540	0.23		
Total	3995.00	16759			
Corrected Total	3972.58	16758			

**Table 5:** Results of four-way ANOVA (diel * lunar * season * tidal) on the number of minutes with buzzes per 10 minutes. The main effects of diel and season, and the interaction effects of diel * lunar, diel * season, diel * tidal, lunar * season, diel * lunar * season, diel * lunar * tidal, diel * season * tidal, lunar * season * tidal and diel * lunar * season * tidal were all significant sources of variability in the number of minutes with buzzes per 10 minutes. Bold numbers indicate significant effects (*P*< 0.05).

Source	Type III Sum of Squares	df	Mean Square	F	*P*
Corrected Model	56.07	218	0.26	4.64	0.000
Intercept	1.72	1	1.72	31.09	0.000
Diel	0.79	4	0.20	3.58	**0.006**
Lunar	0.41	3	0.14	2.44	0.062
Season	2.53	3	0.85	15.23	**0.000**
Tidal	0.34	3	0.11	2.04	0.107
Diel * lunar	2.19	12	0.18	3.29	**0.000**
Diel * season	1.38	12	0.12	2.08	**0.015**
Diel * tidal	1.65	12	0.14	2.48	**0.003**
Lunar * season	0.89	6	0.15	2.69	**0.013**
Lunar * tidal	0.43	9	0.05	0.86	0.559
Season * tidal	0.86	9	0.10	1.71	0.080
Diel * lunar * season	4.42	23	0.19	3.46	**0.000**
Diel * lunar * tidal	3.44	32	0.11	1.94	**0.001**
Diel * season * tidal	2.45	29	0.08	1.52	**0.036**
Lunar * season * tidal	3.64	18	0.20	3.65	**0.000**
Diel * lunar * season * tidal	4.74	41	0.12	2.09	**0.000**
Error	917.14	16540	0.06		
Total	982.00	16759			
Corrected Total	973.20	16758			

### Lunar patterns

**Fig 7:**
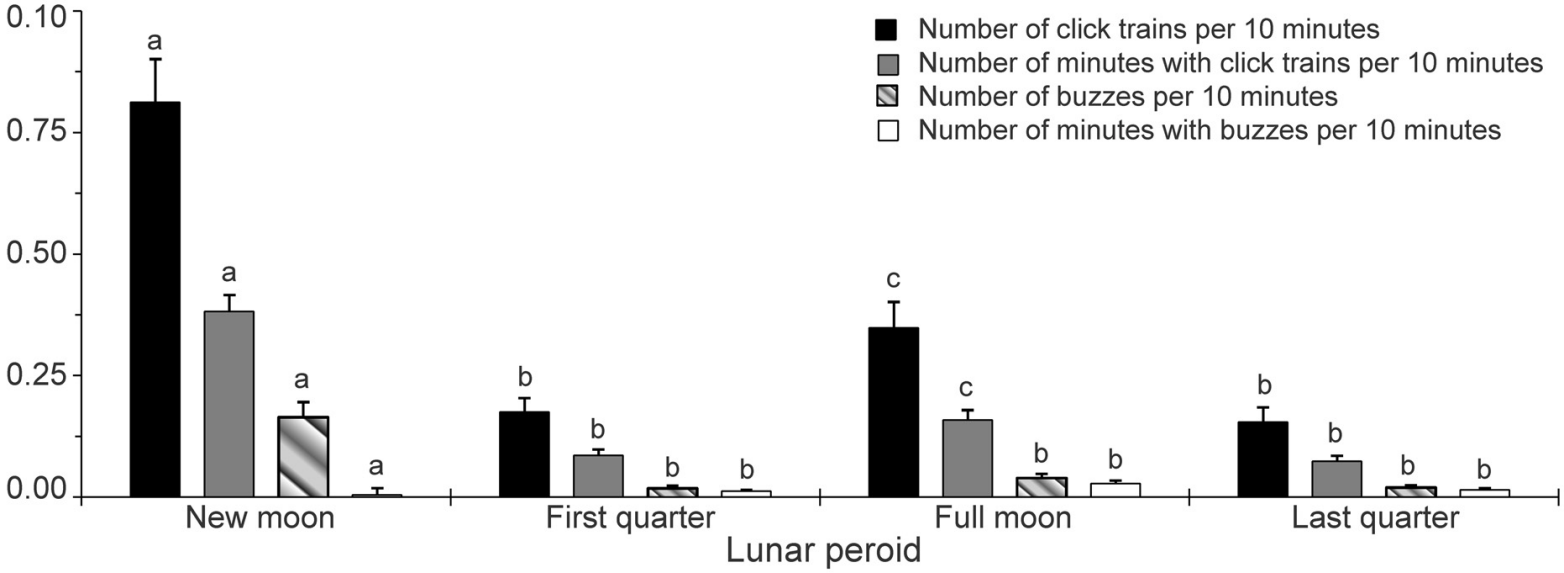
Bar graphs of the number of click trains, number of minutes with click trains, number of buzzes and number of minutes with buzzes per 10 minutes during night time as a function of (A) Diel, (B) lunar, (C) season and (D) tidal. Error bars (mean ± SEM) with different lowercase letters refer to Tamhane’s T2 post hoc multiple-comparison test that yielded significant results *P*< 0.05.

The results of GLM ANOVA indicate that no significant lunar patterns exist in all the four biosonar parameters (Table 2, 3, 4 and 5) (Tamhane’s T2 post hoc multiple-comparison test; *P*> 0.05). However, the subset data indicate that significant lunar patterns were existed in all the biosonar parameters. In particular, at new moon period, both of the number of click trains per 10 minutes and the number of minutes with click trains per 10 minutes were significantly higher than that at the first quarter period, at the full moon period and at the last quarter period, besides, both of those parameters at full moon period was significantly higher than that at the first quarter and the last quarter period (Tamhane’s T2 post hoc multiple-comparison test; *P*< 0.05). Both of the number of buzzes and the number of minutes with buzzes per 10 minutes at the new moon period were significantly higher than that at the first quarter period, and at the full moon period and at the last quarter period (Tamhane’s T2 post hoc multiple-comparison test; *P*< 0.05)(Fig 7). The echolocation encounter durations was significantly different among lunar phases (Kruskal-Wallis χ^2^ = 11.27, df = 3, *P*< 0.01). In particular, echolocation encounter durations during new moon was significantly higher than that at the first quarter (Duncan's multiple-comparison test; *P*< 0.01) (Fig 6B).

### Seasonal patterns

The results of GLM ANOVA indicate that significant seasonal variation was existed in all the four biosonar parameters (Table 2, 3, 4 and 5). Specifically, the number of click trains, the number of minutes with click trains, the number of buzzes and the number of minutes with buzzes per 10 minutes during spring and winter were significantly higher than that during summer and autumn (Tamhane’s T2 post hoc multiple-comparison test; *P*< 0.05) (Fig 5B). Additionally, the echolocation encounter durations were significantly different among seasons (Kruskal-Wallis χ^2^ = 24.50, df = 3, *P*< 0.01). In particular, the echolocation encounter durations during the spring and winter were also significantly higher than that in the autumn (Duncan's multiple-comparison test; *P*< 0.01) (Fig 6C).

### Tidal patterns

The results of GLM ANOVA indicate that no significant differences exist in either parameters of the number of click trains (Table 2), the number of buzzes (Table 4) or the number of minutes with buzzes per 10 minutes among tidal phases (Table 5). Whereas significant differences in the number of minutes with click trains per 10 minutes were observed among tidal phases (Table 3). Specifically, the number of minutes with click trains per 10 minutes in flood tide was significantly lower than that in high tide (Tamhane’s T2 post hoc multiple-comparison test; *P*< 0.05) (Fig 5C). However, the echolocation encounter durations was not significantly different among tidal conditions (Kruskal-Wallis χ^2^ = 1.15, df = 3, *P* = 0.77) (Fig 6D).

## Discussion

A previously published line transect survey indicated that a spatial and temporal segregation in the habitat usages existed in its sympatric Indo-Pacific finless porpoise (*N*. *phocaenoides*), which mainly occur in the southern and eastern water of Hongkong and does not appear to occur in most of Lingding bay of the Pearl River Estuary [42]. No other dolphin species was observed in this region during our historical survey, therefore, species identification was not required in this study.

### Diel variation

The diel pattern in the biosonar activity of humpback dolphins in Pearl River Estuary showed a significantly higher number of echolocation detections at night than that during the day. This pattern could be also widely found in other studies, including the Yangtze finless porpoise in port areas of the Yangtze river, China [25], melon-headed whales (*Peponocephala electra*) at Palmyra Atoll [43], Amazon river dolphins (*Inia geoffrensis*) in the lake and junction at a sustainable development reserve in Brazil [44], harbor porpoises at a re-established stony reef in the northern Kattegat, Denmark [45], near bridge pillars in the Inner Danish Waters between the islands of Zealand and Funen [46], around an offshore gas installations in the Dogger Bank region of the North Sea [26], and in Bloody Bay, West Scotland, UK [38], Heaviside’s dolphins (*Cephalorhynchus heavisidii*) in Walvis Bay, Namibia [47], Risso’s dolphin (*Grampus griseus*) in the Southern California Bight [21], Pacific white-sided dolphin (*Lagenorhynchus obliquidens*) in the Southern California Bight (Type A click bouts) [48], Hawaiian spinner dolphins (*Stenella longirostris*) at Kealakekua Bay, Hawaii [49], common dolphins (*Delphinus delphis*) off the West Wales coast of the British Isles [50], Odontocete (not identified into species) in Onslow Bay, North Carolina [20] and in northeastern Taiwan [51]. The number of click trains in the morning and the evening did not show significant differences from those during the day and at night, suggesting that the morning and evening periods could be inferred as transitional phases between day and night. The significantly higher number of buzzes and number of minutes with buzzes at night suggest that humpback dolphins mainly feed at night. This behavior was also observed in Yangtze finless porpoise in the port of the Yangtze river, China [25] and harbor porpoises around an offshore gas installations in the Dogger Bank region of the North Sea [26]. The diel pattern of humpback dolphins biosonar clicks, feeding buzzes and echolocation encounter duration observed in this study might be ascribed to the temporal variability of their prey as was widely observed in other studies [21, 26, 29, 44, 52]. In addition, these patterns may also associate with the illumination conditions. The higher illumination during the day may allow the dolphins to use both vision and biosonar in locating and identifying objects. Dolphin may have co-processed acoustic and visual information and integrated them in real time [53]. This was further supported by the observations that the active echolocation activity of harbor porpoise increased when ambient lighting was abruptly decreased [54].

### Lunar variations

Lunar periodicity is commonly observed in the reproductive physiology and behavior of marine fish species [28, 55, 56]. Significant higher number of clicks and buzzes detected at the new moon period than at first quarter period, at full moon period, and at last quarter period, may possibly be associated with increased illumination in the later three lunar periods, allowing the dolphins to use both vision and biosonar in locating and identifying fish. This was further supported by the above observed diel pattern with lower echolocation detection during the day.

### Seasonal variation

The higher observation of biosonar activities and with longer echolocation encounter duration in the winter and spring than in the summer and autumn is consistent with the former observations of humpack dolphins in the eastern Pearl River Estuary byline transect survey [23]. Seasonal changes in the distributions of the humpback dolphins were also observed in the Pearl River Estuary [5, 57], in the water of Xiamen [58] and western Taiwan [59]. Dolphins were observed to inhabit an inshore estuary area during winter and spring and shift to offshore areas during summer and autumn [5, 58, 59]. However in the western Pearl River Estuary, an opposite distribution trend occurred [60]. The offshore shifting of the dolphins in the eastern Pearl River Estuary in summer and autumn (wet seasons) may account for the lower biosonar behavior detection in these seasons compared with that during winter and spring (dry seasons). Besides, the observed seasonal variation in the biosonar of humpback dolphins in the Pearl River Estuary might correspond with seasonal abundance and movements of their prey [5]. Humpback dolphins appear to rely almost exclusively on fish for food [61, 62]. They exploit both bottom-dwelling species (e.g., croaker and catfish), and pelagic species (e.g., anchovies and cutlassfishes) [62]. In addition, humpback dolphins were frequently observed feeding at or near seawater/freshwater mixing zones [57]. Their most frequent and important prey in the Pearl River Estuary are the brackish water species of croaker (*Johnius sp*.), lionhead (*Collichthys lucida*) and anchovies (*Thryssa spp*.). During the summer and autumn (wet seasons), the seawater/freshwater mixing zones area were enlarged with an increase of freshwater inflow from the Pearl River. Brackish water species such as lionhead were observed to extend to the offshore side of the estuary, i.e. shifting southward and eastward [22, 63]. However, during winter and spring (dry seasons), an inshore shift was observed [63]. This offshore area occupation during the wet seasons and inshore shift during the dry seasons of fishes coincided well with the same trend of humpback dolphins in this region as revealed by both former visual line transect research [5] and present acoustic monitoring. Significant seasonal variation in the group composition in both of the demersal and shoaling fish were observed in Pearl River Estuary [64]. The local fish species variations maybe linked with their feeding migrations and/or different spawning seasons, e.g. the spawning seasons of lionhead starting in March and lasting until December, whereas, the spawning seasons of *Johnius belengerii* was between May and July [64]. In addition, the fish assemblage variation may be further influenced by their zooplankton and/or phytoplankton prey. The spatial-temporal variability of phytoplankton assemblages under the influence of water turbidity and temperature was observed in the Pearl River estuary [65, 66]. How the environmental parameters impact the local fish stock and further influence the humpback dolphins needs further research.

### Tidal variation

Tidal cycle affecting dolphin acoustic encounters has been widely observed in other species. The biosonar encounters of bottlenose dolphins in the Shannon Estuary, Ireland peaked in ebb tide [67, 68], whereas those in the Moray Firth [69] and in the Clarence River estuary and Richmond River estuary in northern New South Wales, Australia [30] and harbor porpoises in the Bay of Fundy [70] were observed with higher dolphin sightings during flood tide. However, detection of humpback dolphin in Hongkong and at a riverside of an estuary of western Taiwaihad an opposite trend, with significant higher detection in Hongkong and lower detection in Taiwan during ebb tide, respectively [57, 71]. On the contrary, no effects of tidal conditions on the dolphin sighting frequency was observed in humpback dolphins in the offshore side of the same estuary in western Taiwai [71], in bottlenose dolphins in an open water area of Bahía San Jorge, México [72], in Cardigan Bay, West Wales [73] and marine tucuxis (*Sotalia guianensis*) in Guanabara Bay, southeastern Brazil [74] and in the north-eastern coast of Brazil [29]. The tidal cycle affected dolphin sighting, as well as the animal’s behavior was widely attributed the relationship with prey; higher prey availability and/or the lower energy expenditure on obtaining them [29, 30, 67–70, 73–75]. The observed significantly higher number of minutes with click trains at flood tidal conditions than found in high tidal condition in this study might be also a response to their prey distribution.

### Limitations

During the echolocation signal analysis, Pulses within 2 ms after the direct path pulse were eliminated. This was due to the signal processing design of A-tag which is not able to correctly record pulsed sound with inter click intervals shorter than the sampling interval of the A-tag (0.5 ms and 2 ms for short baseline and long baseline, respectively). The high-speed counter of the A-tag employs a 10-bit system (2^10^ = 1024 count). Once a hydrophone was triggered by a pulse, the A-tag waits for the second trigger of the same pulse arrival at the other hydrophone up to 0.277 ms (short baseline: 271 ns * 1024 count) or 1.11 ms (long baseline:1084 ns * 1024 count) in order to measure the time arrival difference correctly. If the inter-click interval between two pulses was shorter than the sampling interval, two pulses passed the hydrophone within one sampling interval of A-tag, but only the highest sound pressure level (either from the first pulse or the second pulse) would have been logged by the A-tag. In addition, another trigger by the second pulse may even happened before the second trigger of the first pulse happened at the other hydrophone and triggered the A-tag to have started a new measurement of the time difference. Additional limitation such as duplicate acoustic detections of the same animal and/or underestimating the low level clicks and buzzes, and non-vocal animals can be referenced in detailed in reference [25, 31].

### A-tag acoustic detection range

**Fig 8:**
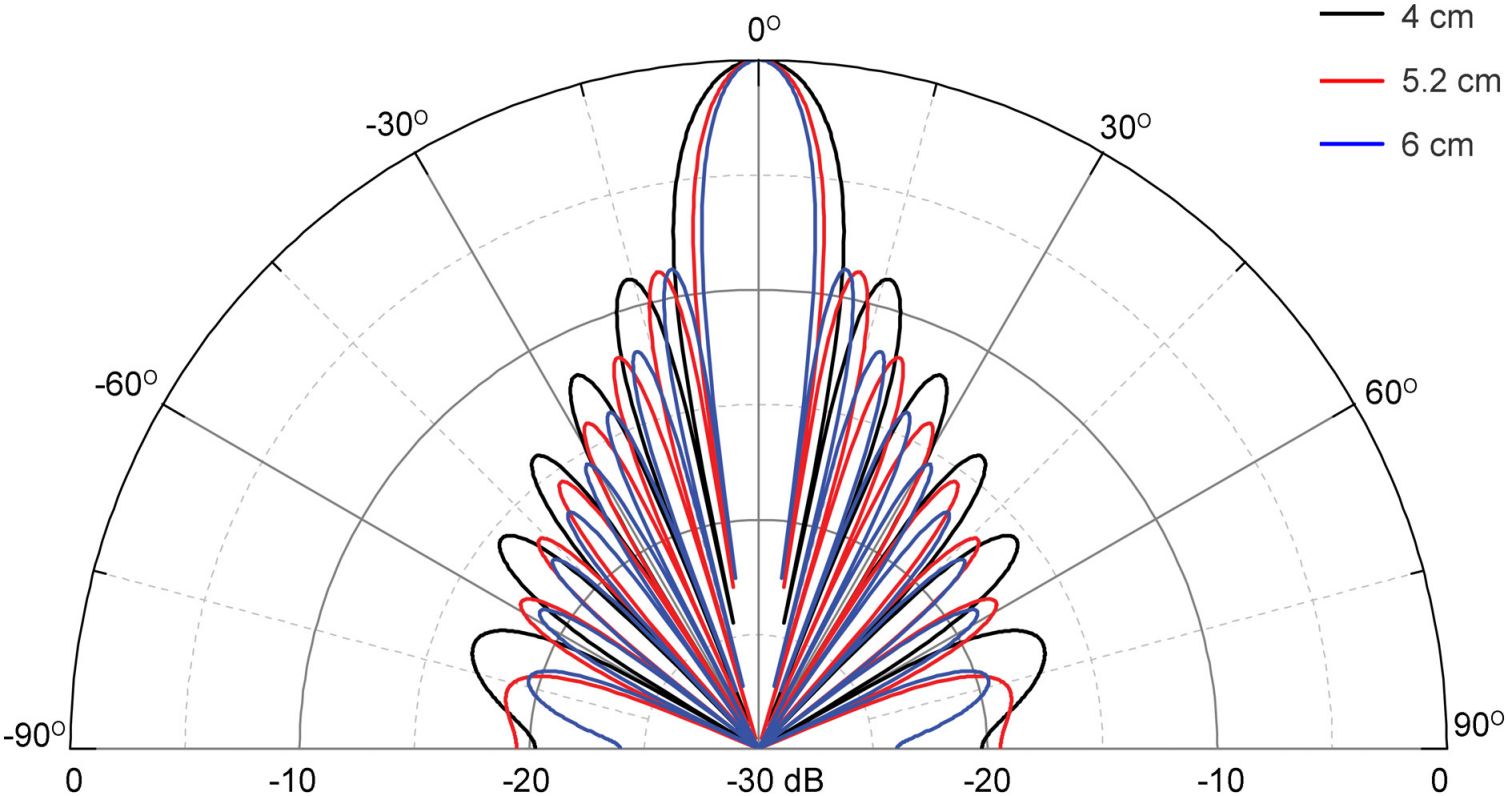
Beam pattern of circular piston transducer. Modeling was based by using a radius of 4 cm (directivity index = 25.6 dB), 5.2 cm (directivity index = 27.9 dB) and 6 cm (directivity index = 29.2 dB) piston transducer at typical peak frequency of humpback dolphin echolocation click of 114 kHz.

Factors that may affect acoustic detection include the intensity and directionality of the sound source, sound propagation conditions, acoustic masking by the ambient noise and the characteristics of the acoustic detector, such as the self-noise of the system. The signal to noise ratio (SNR) of a humpback dolphin echolocating click at the receiver can be estimated by application of the passive sonar equation [76]:

SNR = SL−DI−TL−NL (3)

$$TL\;=\;k\;{\text{log}}_{10}r+ar$$ (4)

where SL is the dolphin’s on-axis source level (measured in dB re 1μPa at 1 m), and the humpback dolphins emitted biosonar clicks with a mean apparent source level of 199 ± 3 dB re 1μPa peak-peak (range: 194 dB –208 dB) [17], *DI* is the directivity index of the on-axis signal at different angle from the animal’s acoustic axis; *NL* is the combined function of the ambient noise level (measured in dB re 1μPa) and the electronic self-noise of the recorder, *TL* is sound transmission loss as a function of distance (*r*, in m) between sound source (dolphin) and receiver, *k* is the environment–dependent transmission loss coefficient, which normally ranges from spherical spreading loss (k = 20) to cylindrical spreading loss (k = 10) [76], *a* is frequency-dependent absorption coefficient and was estimated at 0.036 dB/m for clicks with peak frequency of 114 kHz for sea water of salinity 35‰ and pH = 8.0 as a function of site specific pressure and temperature (with the lowest and highest temperature averaged at 13.59°C and 30.63°C in January and August, respectively) [66] at Pearl River Estuary according to the Fisher and Simmons equation [77]. Information concerning the empirical measurements of the directivity index and beam pattern for humpback dolphins is currently unavailable. However, a circular piston transducer model was formerly assumed as an approximation of the odontocetes sound source and the directivity index of bottlenose dolphin echolocating clicks matched well with a 4 cm radius circular piston projector [32], pistons with an equivalent piston radius of 5.2 cm gave the best fit with the Atlantic spotted dolphins (*Stenella frontalis*) on-axis echolocating clicks [78], whereas pistons with radii of 6 cm gave the best fit with the main beam of white-beaked dolphin (*L*. *albirostris*) [79] and false killer whale (*Pseudorca crassidens*) [80] echolocating clicks. Hence, circular piston with an radii of 4 cm, 5.2 cm and 6 cm were adopted to theoretically calculate the beam pattern and directivity index of humpback dolphins echolocating clicks according to the equations [32]:

$$BP=\text{1}0\times {\text{log}}_{10}|2\times \text{besselj(}1\text{, }k\times \text{sin}(\theta )\text{)/(}k\times \text{sin}(\theta ))|$$ (5)

$$DI=10\times {\text{log}}_{10}[k^{2}/(1-\text{besselj(}1\text{, }2\times k\text{)/}k)]$$ (6)

k = 2×π×f×a/c (7)

where ‘besselj’ is the Bessel function of the first kind, f is set at the peak frequency of humpback dolphin echolocating clicks of 114 kHz [17], and a is the piston radius in m. The modeled beam pattern has a directivity index of 25.6 dB, 27.9 dB and 29.6 dB for a 4 cm, 5.2 cm and 6 cm circular piston model, respectively (Fig 8). Since a hardware detection threshold (DT) of 135.7 dB was adopted by the A-tag in this study, the passive sonar equation could be modified as:

SNR = SL−DI−TL−max(NL, DT)  (8)

In situations where the noise level is lower than the detection threshold of the A-tag, i.e. the masking effect by noise can be ignored, the average detection range of the humpback dolphin echolocation clicks with a mean apparent source level of 199 dB was estimated at 600 m (on animal’s acoustic axis) and 100 m (off the animal’s acoustic axis with a directivity index of 29.6 dB), respectively after accounting for an intermediate transmission loss of 15log_10_ *r*. Click with source level of 208 dB can be detected at a maximum range of 1200 m on the animal’s acoustic axis after accounting for a cylindrical spreading loss of 10log_10_ *r* and signals with a source level of 194 dB can be detected at a maximum range of 25 m off the animal’s acoustic axis after accounting for a spherical spreading loss of 20log_10_ *r*. However, these are only theoretical results and need be further corroborated by empirical measurements.

### Artificial reef effect

The underwater structures of some man-made platforms and installations may have acted as artificial reefs and may have influenced local biodiversity and provide consequences to the marine ecosystem. These structures can attract large aggregations of plankton, epifaunal communities, lead to colonization via hydrodynamic effects [81, 82], and/or provide shelter for a variety of marine organisms against currents or predators—consequently lead to locally increased biomass and enhanced biodiversity [83, 84]. Besides, all types of fisheries are prohibited and excluded in these man-made platforms or installations, leading to less human disturbance of the local fish aggregations [84]. Whether the signal tower in this study also acting some artificial reef effect as observed in other studies [46, 85, 86] deserve future research. Multiple passive acoustic monitoring devices deploy at a graded distance from the signal tower could shed light on this issue.

## Conclusions

A long-term static passive acoustic monitoring strategy was applied in the investigation of the presence of the humpback dolphins in the Pearl River Estuary. The possible diel, lunar, seasonal and tidal patterns in their biosonar activities were analyzed through a Generalized Linear Model approach. The results revealed significant diel, seasonal, and tidal patterns. The biosonar activities (both of the detection of echolocation click and feeding buzzes) at night were significantly higher than that during the day, indicating that humpback dolphins mainly feed at night in this region. Significantly higher biosonar behavior at high tide than at flood tide, and in winter-spring than in summer-autumn was also observed. The lunar patterns was evident for the dolphin biosonar behavior at night time, and the diel, lunar and seasonal patterns for the echolocation encounter duration were also significantly varied. All these diel, lunar, seasonal, and tidal pattern in the humpback dolphin biosonar behaviors in Pearl River Estuary may be due to the spatial-temporal variability of their prey and visibility in the water. The baseline data of the dolphin distribution in this region before the construction should help in the design of effective mitigation programmes before construction. For example, perhaps construction should be avoided during the periods with higher dolphin presence, such as at night and during the winter and spring seasons. This baseline information can further facilitate later assessments of the effects of offshore wind farms on the local dolphins by providing data for future comparison.

Since habitat use of some individual humpback dolphins in Hongkong waters showed an obvious variation among years [22], longer-term and range-wide monitoring of the humpback dolphins as well as local fish distributions will be necessary to elucidate the annual and spatial variation regarding dolphin presence and activities as well as the possible parameter that trigger this pattern in the Pearl River Estuary. This relevant information can help the protection of the local dolphin species and drive further action on the managing of anthropogenic impacts in light of increasing threats from infrastructure development projects within the distribution range of this species.
